# Effect of CXCR5, PD‐1 and ICOS on B‐cell responses and relevance to myasthenia gravis

**DOI:** 10.1002/cti2.70112

**Published:** 2026-06-21

**Authors:** Merve Çebi, Arman Çakar, Hacer Durmuş, Yeşim Parman, Güher Saruhan‐Direskeneli

**Affiliations:** ^1^ Department of Physiology, Istanbul Medical Faculty Istanbul University Istanbul Turkey; ^2^ Department of Neurology, Istanbul Medical Faculty Istanbul University Istanbul Turkey; ^3^ Present address: M Çebi, Department of Medical Biology School of Medicine, Recep Tayyip Erdoğan University Rize Turkey

**Keywords:** CXCR5, ICOS, myasthenia gravis, PD‐1, T follicular helper cells, T peripheral helper cells

## Abstract

**Objectives:**

T follicular helper (Tfh) cells regulate B‐cell responses within germinal centres primarily via programmed cell death protein 1 (PD‐1) and inducible T‐cell costimulator (ICOS), whereas CXC chemokine receptor type 5 (CXCR5)‐negative T peripheral helper (Tph) cells provide similar support extra‐follicularly. Both subsets contribute to the pathogenesis of acetylcholine receptor‐antibody‐positive myasthenia gravis (AChR‐MG). This study investigated the roles of PD‐1, ICOS and CXCR5 in T‐cell‐mediated B‐cell activation to identify therapeutic targets for MG.

**Methods:**

CD4 T cells from 10 healthy controls and six untreated AChR‐MG patients were sorted by CXCR5, PD‐1 and ICOS expression, stimulated with anti‐CD3/CD28, and co‐cultured with autologous CD19 B cells. Plasmablast differentiation, total AChR‐IgG and cytokine productions were measured.

**Results:**

In healthy donors, PD‐1^+^ or ICOS^+^ Tph cells modestly enhanced plasmablasts and IgG production, while CXCR5^+^ Tfh cells co‐expressing PD‐1 or ICOS induced significantly stronger B‐cell responses. In AChR‐MG patients, PD‐1 expressing T cells promoted plasmablasts, antibody production and cytokine secretion regardless of CXCR5, with amplified effects in CXCR5^+^ co‐cultures.

**Conclusions:**

PD‐1 expression on CD4^+^ T cells is associated with increased B‐cell helper capacity. This effect is more pronounced in Tfh‐related contexts and is linked to dysregulated humoral immune responses in MG. PD‐1 appears to play a central role in the pathogenesis of MG, primarily through Tfh cell activity.

## Graphical abstract

This study compared the efficacy of PD‐1 and ICOS molecules in antibody production within CXCR5^+^ and CXCR5^−^ CD4^+^ T cells; it demonstrates that PD‐1^+^ T cells provide the most effective B cell support, a finding also validated in autoantibody production in myasthenia gravis.

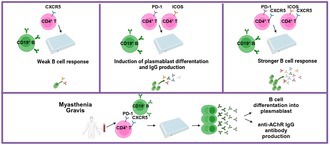

## Introduction

Myasthenia gravis (MG) is an autoimmune disease caused by antibodies against antigens at the neuromuscular junction and characterised by muscle weakness.^1^, ^2^ Autoantibodies play a dominant role in the pathogenesis of MG. In most patients, antibodies develop against the acetylcholine receptor (AChR), and these patients are classified as AChR‐MG.^2^, ^3^ Antibodies against AChR, primarily of the IgG1 and IgG3 isotypes, trigger complement activation, leading to postsynaptic membrane damage, AChR loss and disruption of neuromuscular signal transmission.^4^

T follicular helper (Tfh) cells are a subset of T cells characterised by the expression of surface molecules such as CXC chemokine receptor type 5 (CXCR5), programmed cell death protein 1 (PD‐1) and inducible T‐cell costimulator (ICOS). They primarily secrete IL‐21, along with IL‐4 and IL‐17A. These cells of the germinal center in lymphoid tissue help B cells in activation, differentiation and antibody production.^5^

Recent studies indicate that CXCR5‐lacking CD4^+^ T cells, characterised by elevated IL‐21 secretion and high ICOS and PD‐1 expression, play a significant role in antibody production in autoimmune diseases such as rheumatoid arthritis (RA) and systemic lupus erythematosus (SLE).^6^, ^7^ This population of cells is referred to as T peripheral helper (Tph) cells. Unlike Tfh cells, Tph cells lack expression of CXCR5, but have high levels of receptors such as C‐C chemokine receptors type 2 (CCR2), type 5 (CCR5) and CX3C chemokine receptor 1 (CX3CR1).^8^, ^9^, ^10^ While Tfh cells use CXCR5 to migrate to the germinal center and interact with B cells for long‐term antibody responses, Tph cells express CCR2 to migrate to inflamed tissues and interact with B cells in these areas, contributing to localised antibody responses.^8^ Like Tfh cells, they also exhibit high expression of PD‐1 and ICOS. Despite the well‐established roles of ICOS and PD‐1 molecules in helper T cells' involvement in B‐cell maturation and antibody production,^11^, ^12^, ^13^ the relative effectiveness of these molecules and their precise contributions compared to one another remain unclear. In our previous study, CD4^+^ T‐cell populations resembling Tph (CXCR5^−^PD‐1^+^ or CXCR5^−^ICOS^+^) had higher frequencies in the blood of MG patients, similar to Tfh (CXCR5^+^PD‐1^+^ or CXCR5^+^ICOS^+^) cell populations.^14^ The aim of this study is to comprehensively assess the roles of T cells expressing CXCR5, ICOS, and/or PD‐1 molecules in B‐cell differentiation and antibody production, and to compare the effects of Tfh and Tph cell populations on B‐cell responses in terms of plasmablast generation and antibody production.

## Results

### Isolated effects of CXCR5, PD‐1 and ICOS molecules of helper T cells on B‐cell responses

To understand the differential effects of CXCR5, ICOS and PD‐1 molecules of helper T cells on B cells, co‐cultures from healthy controls (HC) containing helper T cells expressing only one of these molecules (CXCR5^+^ICOS^−^PD‐1^−^, PD‐1^+^CXCR5^−^ICOS^−^, ICOS^+^CXCR5^−^PD‐1^−^) were compared for the induction of plasmablasts (PB) and the production of total IgG.

**Figure 1:**
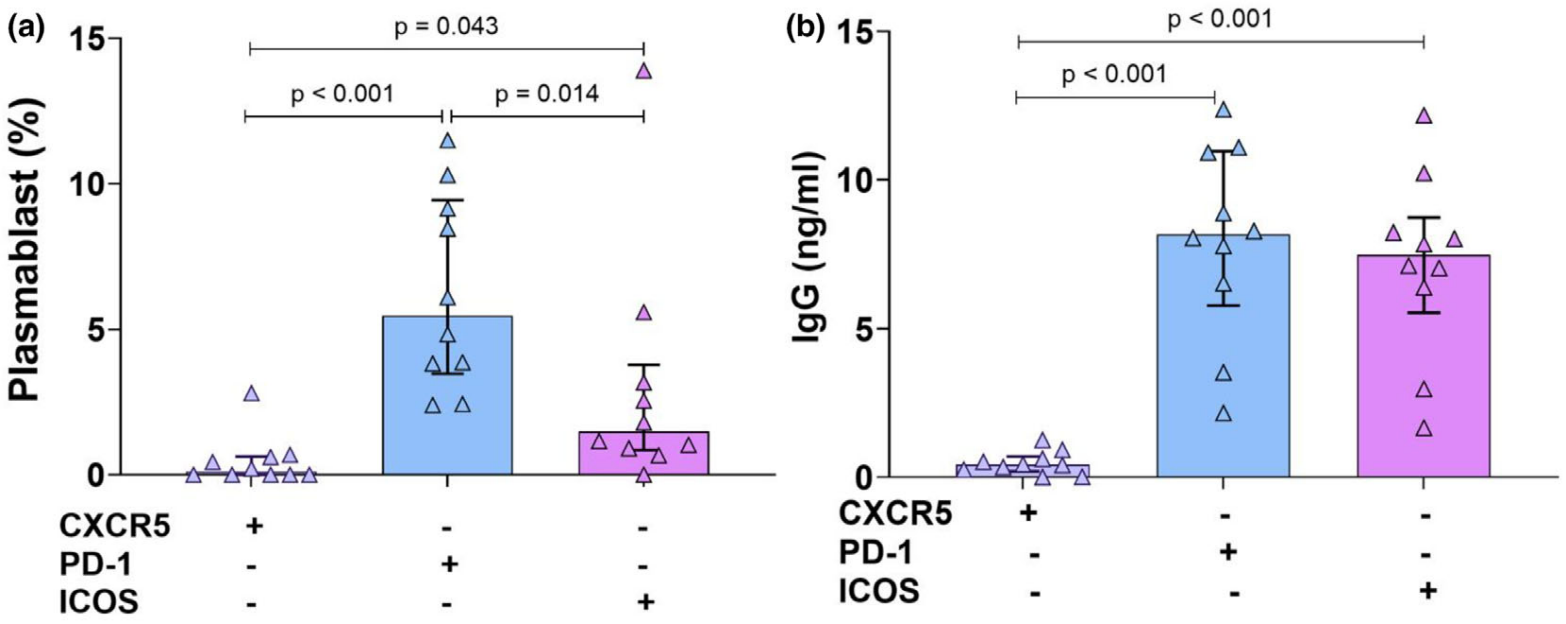
Impact of CXC chemokine receptor type 5 (CXCR5), programmed cell death protein 1 (PD‐1) and inducible T‐cell costimulator (ICOS) molecules in co‐cultures. Comparison of plasmablasts (CD27^+^CD38^+^ in CD19^+^ cells, PB) **(a)** and IgG concentrations **(b)** in co‐cultures of helper T cells expressing only CXCR5, PD‐1 or ICOS with B cells. Co‐cultures were performed once per condition using cells from healthy donors (*n* = 10). PD‐1^+^ (*P* < 0.001) or ICOS^+^ (*P* = 0.043) helper T cells showed increased plasmablasts (PB) and IgG levels (both *P* < 0.001) compared with CXCR5^+^ cells. PB levels were higher in PD‐1^+^ compared with ICOS^+^ co‐cultures (*P* = 0.014). No technical replicates were performed. Data are presented as medians with interquartile ranges. Statistical analyses were performed using non‐parametric tests with *P* < 0.05 considered significant.

When evaluating populations across co‐cultures, the PB were significantly higher in co‐cultures containing helper T cells expressing only PD‐1 (*P* < 0.001) or only ICOS (*P* = 0.043) than in those with helper T cells expressing only CXCR5 (Figure 1a). Additionally, helper T cells expressing only PD‐1 have also induced higher proportions of PB in co‐cultures than in those with helper T cells expressing only ICOS (Figure 1a, *P* = 0.014).

When assessing the total IgG concentrations in supernatants, co‐cultures with helper T cells both expressing only PD‐1 (*P* < 0.001) or only ICOS (*P* < 0.001) revealed elevated IgG concentrations compared to those with T cells expressing only CXCR5 (Figure 1b). In contrast to the co‐culture conditions, control cultures containing only CD19^+^ B cells demonstrated no plasmablast induction and IgG production in the absence of helper T cells (data not shown).

### The PD‐1 and ICOS molecules on helper T cells are more effective with CXCR5^+^

**Figure 2:**
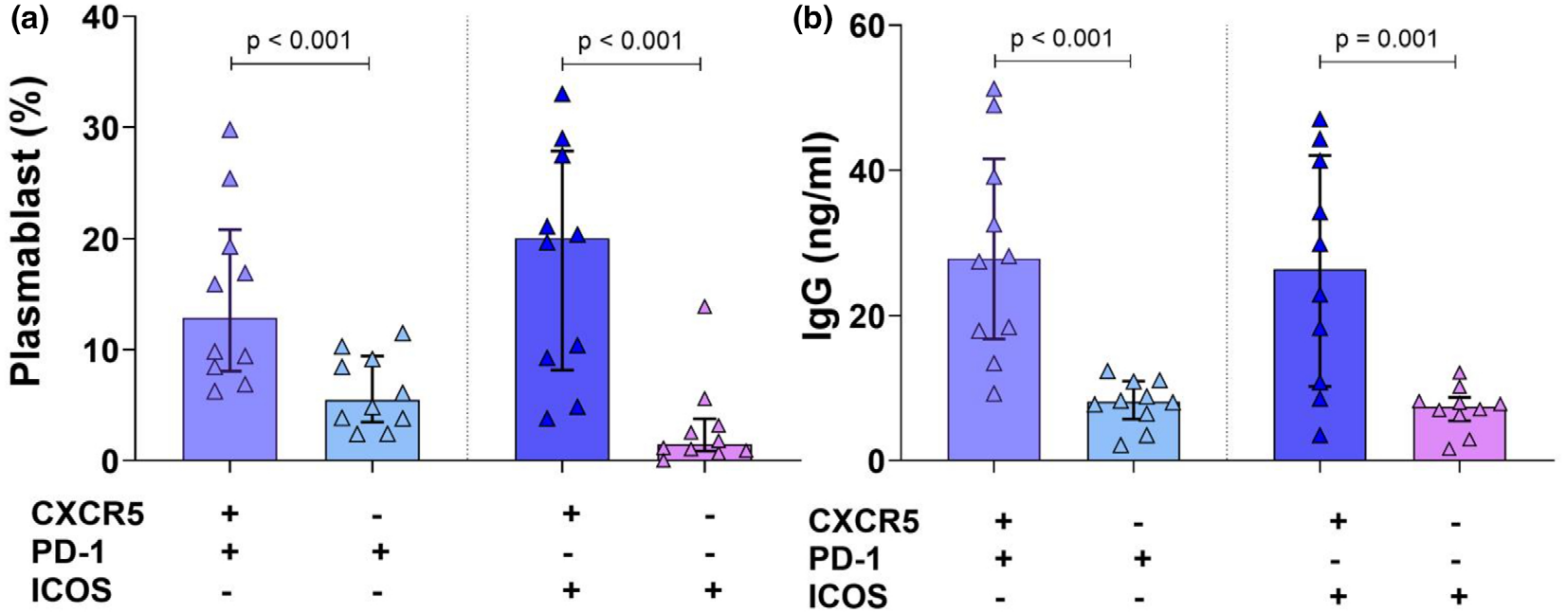
Comparative analysis of programmed cell death protein 1 (PD‐1) and inducible T‐cell costimulator (ICOS) effects on B‐cell responses in CXCR5^+^ vs. CXCR5^−^ helper T cells. Both co‐cultures with PD‐1^+^ (*P* < 0.001 both) or ICOS^+^ (*P* < 0.001 and *P* = 0.001) in addition to CXCR5^+^ helper T cells showed increased plasmablast populations **(a)** and higher IgG levels **(b)** compared with those with CXCR5^−^ helper T cells. Healthy controls (HC) (*n* = 10). Data are presented as medians with interquartile ranges. Statistical analyses were performed using non‐parametric tests with *P* < 0.05 considered significant. CXCR5, CXC chemokine receptor type 5.

The common feature of both Tfh and Tph cells, playing active roles in B‐cell responses, is their high expression of PD‐1 and ICOS. However, Tph cells do not express CXCR5 like Tfh cells. To evaluate the effects of Tfh and Tph cells on B‐cell responses, co‐cultures containing CXCR5 carrying helper T cells were compared with co‐cultures without CXCR5 helper T cells. Higher PB (Figure 2a) and IgG concentrations (Figure 2b) were observed in co‐cultures of PD‐1 (both *P* < 0.001) or ICOS (both *P* < 0.0001) carrying CXCR5^+^ helper T cells compared to CXCR5^−^ helper T‐cell co‐cultures.

### In AChR‐MG patients PD‐1 expressing T cells with or without CXCR5 induce effective B‐cell responses

Although the expression of ICOS is also reported in both Tfh and Tph cells,^10^ Tfh cells are typically characterised as CXCR5^+^PD‐1^+^ and Tph cells as CXCR5^−^PD‐1^+^. Moreover, PD‐1 expressing cells induced higher plasmablast populations and antibody production in the HC in this study (Figure 1a). Based on these findings, the effects of CXCR5^+^PD‐1^+^, CXCR5^−^PD‐1^+^, CXCR5^−^PD‐1^+^ and CXCR5^−^PD‐1^−^ cell populations, all without ICOS, were evaluated in co‐cultures for development of PB and IgG concentrations in AChR‐MG patients.

**Figure 3:**
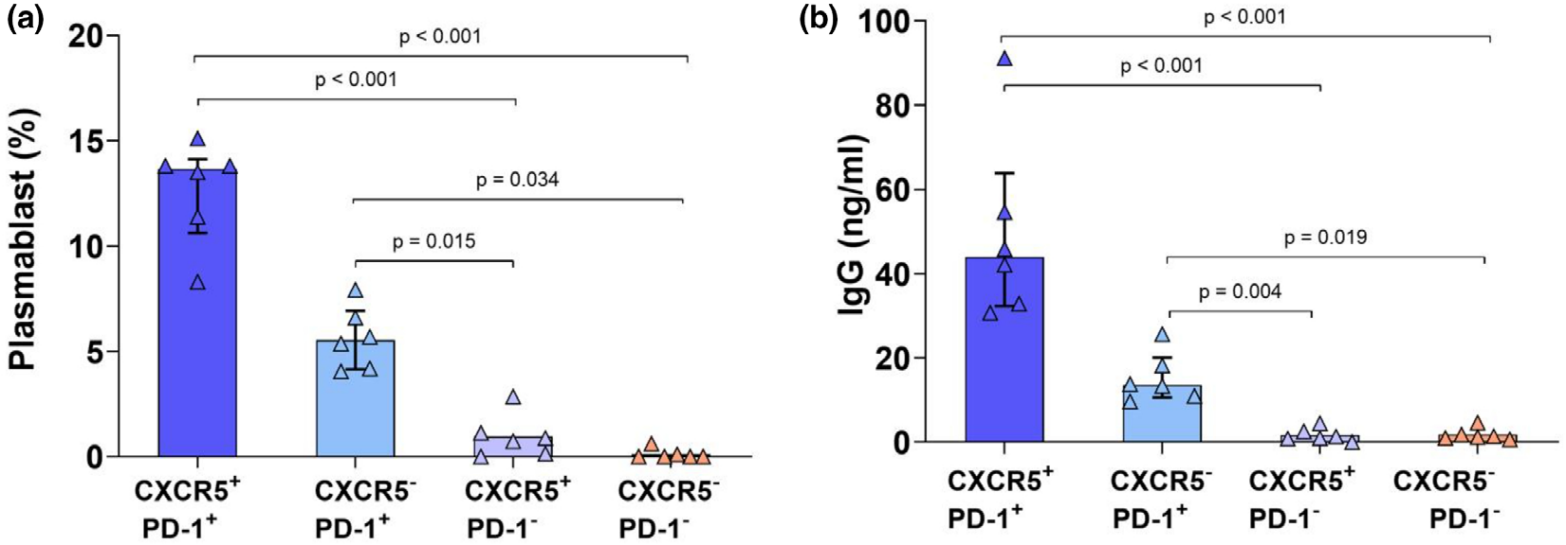
Plasmablast and IgG levels in co‐cultures of acetylcholine receptor‐antibody‐positive myasthenia gravis (AChR‐MG) patients. In AChR‐MG patients (MG, *n* = 6), co‐cultures including CXCR5^+^PD‐1^+^ (both *P* < 0.001) and CXCR5^−^PD‐1^+^ (*P* = 0.015 and *P* = 0.034) helper T cells displayed markedly higher plasmablast frequencies **(a)** than those with CXCR5^+^PD‐1^−^ or CXCR5^−^PD‐1^−^ cells (all ICOS^−^). Similarly, IgG levels **(b)** in CXCR5^+^PD‐1^+^ (both *P* < 0.001) and in CXCR5^−^PD‐1^+^ co‐cultures (*P* = 0.004 and *P* = 0.019) were higher than those with CXCR5^+^PD‐1^−^ or CXCR5^−^PD‐1^−^ cells. Experiments were performed once per condition without technical replicates. Statistical analyses were performed using non‐parametric tests with *P* < 0.05 considered significant. Data are represented as medians with interquartile ranges. CXCR5, CXC chemokine receptor type 5; ICOS, inducible T‐cell costimulator; PD‐1, programmed cell death protein 1.

In AChR‐MG patients (MG, *n*: 6), co‐cultures including CXCR5^+^PD‐1^+^ (both *P* < 0.001) and CXCR5^−^PD‐1^+^ (*P* = 0.015 and *P* = 0.034) helper T cells displayed markedly higher plasmablast frequencies than those with CXCR5^+^PD‐1^−^ or CXCR5^−^PD‐1^−^ cells (Figure 3a). Similarly, IgG levels in CXCR5^+^PD‐1^+^ (both *P* < 0.001) and in CXCR5^−^PD‐1^+^ co‐cultures (*P* = 0.004 and *P* = 0.019) were higher than those with CXCR5^+^PD‐1^−^ or CXCR5^−^PD‐1^−^ cells (Figure 3b).

**Figure 4:**
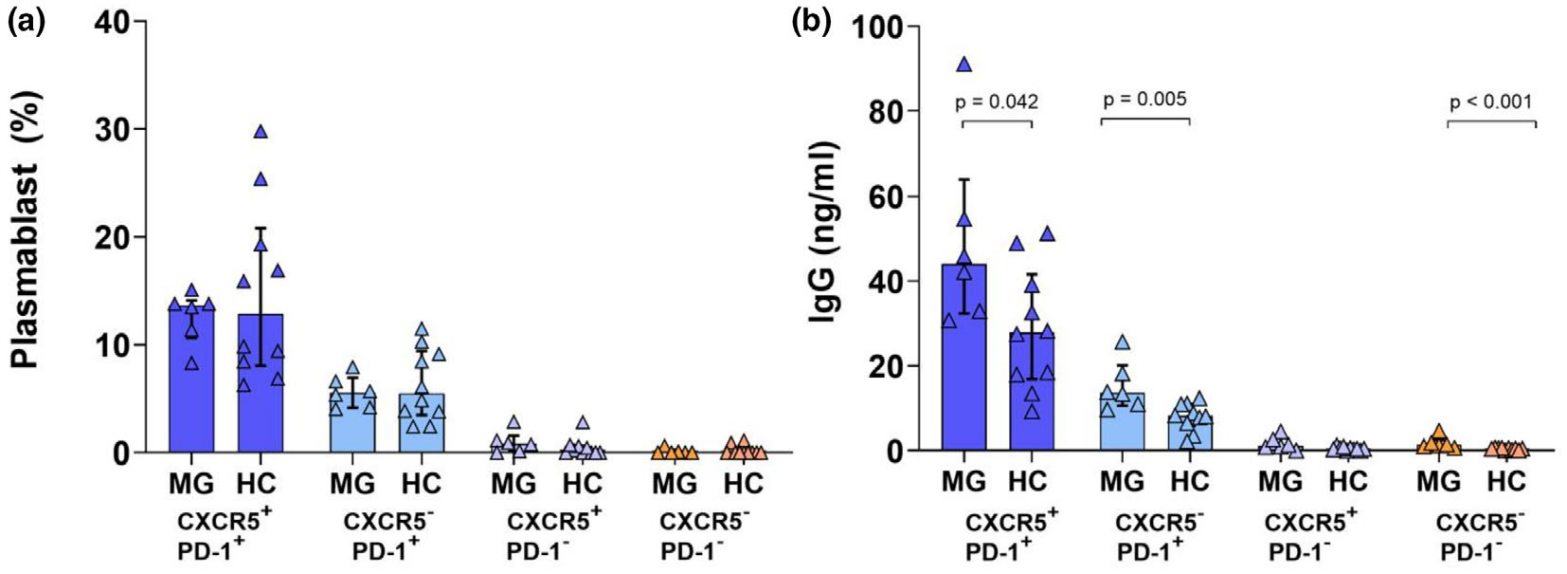
Comparison of plasmablast and IgG levels between acetylcholine receptor‐antibody‐positive myasthenia gravis (AChR‐MG) patients and healthy controls (HC). **(a)** Plasmablast populations were not different between groups. **(b)** IgG levels were higher in co‐cultures with CXCR5^+^PD‐1^+^ (*P* = 0.042) and CXCR5^−^PD‐1^+^ (*P* = 0.005) helper T cells from AChR‐MG patients compared with HC. IgG secretion was also higher in the AChR‐MG patient group, when CXCR5^−^PD‐1^−^ helper T cells are compared (*P* < 0.001). Data are presented as medians with interquartile ranges. Experiments were performed once per condition without technical replicates, using independent patient and control samples (MG *n* = 6, HC *n* = 10). Statistical analyses were performed using non‐parametric tests with *P* < 0.05 considered significant. CXCR5, CXC chemokine receptor type 5; PD‐1, programmed cell death protein 1.

Subsequently, PB and IgG concentrations in co‐cultures were compared between HC and AChR‐MG patients. In the AChR‐MG patient group, higher IgG secretion was observed in CXCR5^+^PD‐1^+^ (*P* = 0.042) and CXCR5^−^PD‐1^+^ (*P* = 0.005) helper T‐cell co‐cultures compared to HC (Figure 4b). IgG secretion was also higher in the AChR‐MG patient group when CXCR5^−^PD‐1^−^ helper T cells are compared (*P* < 0.001). The proportions of the PB were not different between groups (Figure 4a).

### CXCR5 ^+^ PD‐1^+^ helper T cells are key contributors to anti‐AChR‐IgG production

**Figure 5:**
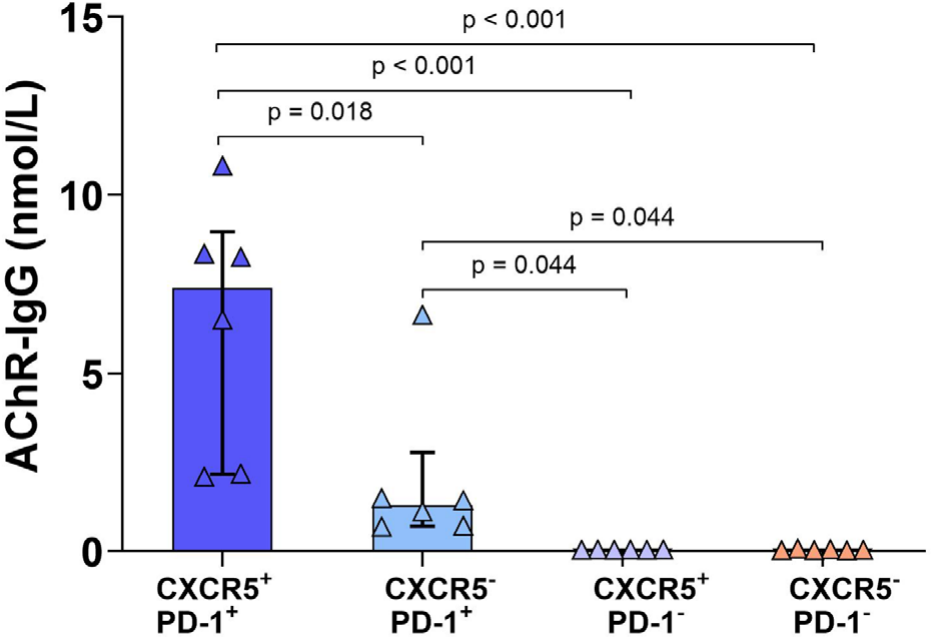
Anti‐AChR‐IgG production in co‐cultures from acetylcholine receptor‐antibody‐positive myasthenia gravis (AChR‐MG) patients. AChR‐IgG concentrations in the supernatants of CXCR5^+^PD‐1^+^ helper T‐cell co‐cultures were significantly higher than those observed in CXCR5^−^PD‐1^+^ (*P* = 0.018), CXCR5^+^PD‐1^−^ and CXCR5^−^PD‐1^−^ cell co‐cultures (both *P* < 0.001). AChR‐IgG levels in CXCR5^−^PD‐1^+^ helper T‐cell co‐cultures were also elevated compared with CXCR5^+^PD‐1^−^ and CXCR5^−^PD‐1^−^ cell co‐cultures (both *P* = 0.044). Data are presented as medians with interquartile ranges. Experiments were performed once per condition without technical replicates (MG *n* = 6). Statistical analyses were performed using non‐parametric tests, with *P* < 0.05 considered significant. CXCR5, CXC chemokine receptor type 5; PD‐1, programmed cell death protein 1.

As shown above, PD‐1 expressing helper T cells provided an inductive role in B‐cell responses, regardless of whether they were CXCR5 positive. However, this effect was stronger with CXCR5^+^PD‐1^+^ helper T cells. To determine which population was more effective in autoantibody production in MG pathogenesis, specific anti‐AChR‐IgG concentrations were evaluated in co‐cultures of helper T cells from AChR‐MG patients. The AChR‐IgG concentrations in the supernatants of CXCR5^+^PD‐1^+^ helper T‐cell co‐cultures were significantly higher than those with CXCR5^−^PD‐1^+^ (*P* = 0.018), CXCR5^+^PD‐1^−^ and CXCR5^−^PD‐1^−^ cells (both *P* < 0.001) (Figure 5). However, AChR‐IgG levels were also elevated in co‐cultures with CXCR5^−^PD‐1^+^ helper T cells compared to those with CXCR5^+^PD‐1^−^ and CXCR5^−^PD‐1^−^ T cells (both *P* = 0.044) (Figure 5), implicating the importance of PD‐1 even in the absence of CXCR5 which is not observed for CXCR5 alone.

### IL‐4 and IL‐17 levels are increased in PD‐1 expressing T‐cell co‐cultures from AChR‐MG patients

**Figure 6:**
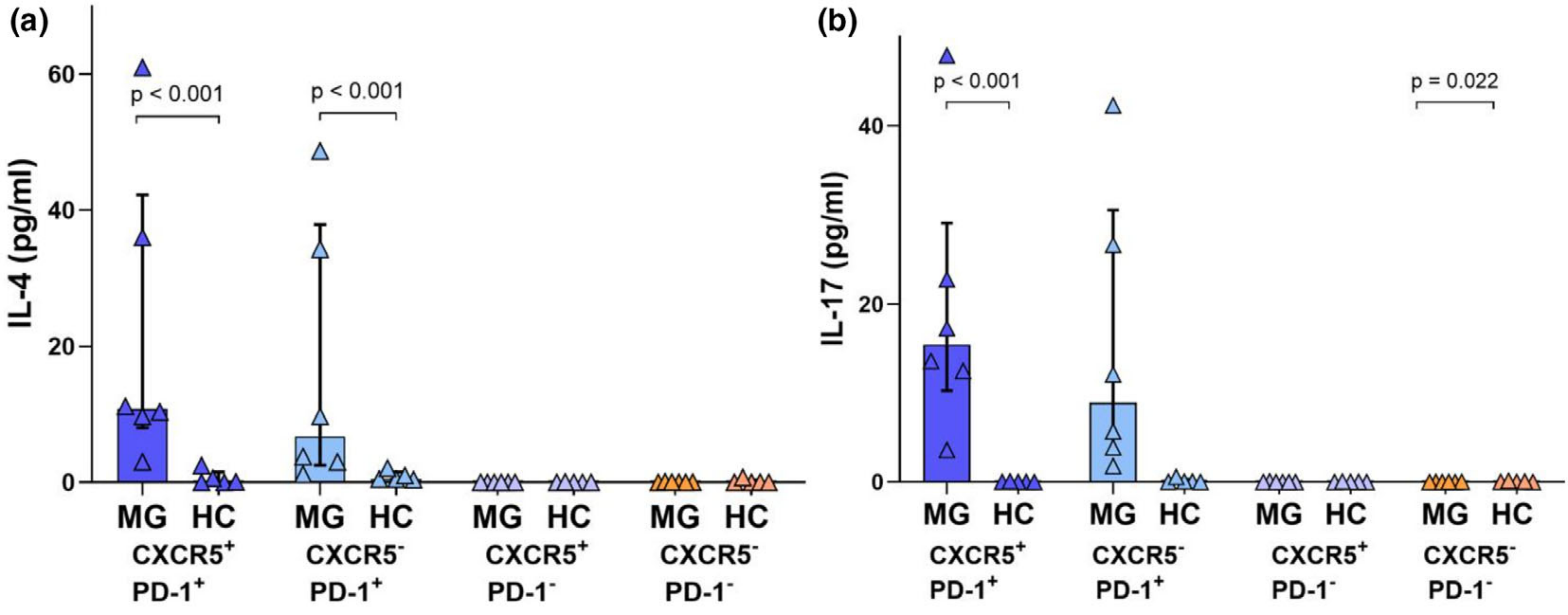
IL‐4 and IL‐17 productions in co‐cultures from acetylcholine receptor‐antibody‐positive myasthenia gravis (AChR‐MG) patients and healthy controls (HC). **(a)** IL‐4 and **(b)** IL‐17 levels were measured in culture supernatants of the co‐cultures. Both IL‐4 and IL‐17 secretions were significantly increased in CXCR5^+^PD‐1^+^ (both *P* < 0.001) helper T‐cell co‐cultures from AChR‐MG patients when compared with HC. CXCR5^−^PD‐1^+^ T cells of patients produced higher IL‐4 levels as well (*P* < 0.001). IL‐17 production was higher in CXCR5^−^PD‐1‐ co‐culture of patients (*P* = 0.022). Data are presented as medians with interquartile ranges. Experiments were performed once per condition without technical replicates (MG *n* = 6, HC *n* = 10). Statistical analyses were performed using non‐parametric tests with *P* < 0.05 considered significant. CXCR5, CXC chemokine receptor type 5; PD‐1, programmed cell death protein 1.

Our previous study identified elevated levels of IL‐21, IL‐17 and IL‐4 in CD4^+^ T cells of MG patients which were attributed to high frequencies of Tfh and Tph cells.^14^ A subsequent study also demonstrated that IL‐21, IL‐17 and IL‐4 were higher in CXCR5^+^ICOS^+^ helper T cells in MG patients than in CXCR5^+^ICOS^−^ cells and HC.^15^ To further delineate cytokine production in MG, cytokine levels were measured with enhanced sensitivity in co‐cultures from AChR‐MG patients and HC with subsets according to CXCR5 and PD‐1 expressing T cells. IL‐21 was not detectable in the supernatants with available kits. IL‐4 and IL‐17 production were higher in CXCR5^+^PD‐1^+^ (both *P* < 0.001) helper T‐cell co‐cultures from AChR‐MG patients compared to HC (Figure 6a and b). CXCR5^−^PD‐1^+^ helper T cells also produced higher IL‐4 in AChR‐MG patients (*P* < 0.001), whereas IL‐17 increase in this comparison was not significant. However, IL‐17 was detected higher in patients' CXCR5^−^PD‐1^−^ helper T‐cell cultures as well (*P* = 0.022).

## Discussion

In the present study, the role of CXCR5, ICOS and PD‐1 molecules on helper T cells is compared for their effects on B‐cell functions, mainly on plasmablast differentiation and antibody production. Their single presence vs. combinational effects, as in Tfh and Tph cells, are compared in HC as well as in AChR‐MG patients for antibody secreting cell development and antigen‐specific or total IgG production. Although the strongest effects of the T cells are detected in cultures containing PD‐1 with CXCR5, referring to Tfh cells, PD‐1 or to a weaker extent ICOS molecules alone also provided the T‐cell help in HC. In AChR‐MG patients, PD‐1 with or without CXCR5 on T cells induced higher antigen‐specific autoantibodies and IL‐4 and IL‐17 secretions.

The study elucidated the role of CXCR5, ICOS and PD‐1 molecules on helper T cells, in the context of an autoimmune disease associated with autoantibodies, specifically in the pathogenesis of AChR‐MG. The involvement of ICOS, PD‐1 and CXCR5 molecules of CD4^+^ T cells in antibody production is well described. In earlier studies, Tfh cells defined as CD4^+^CXCR5^+^ cells were found in both lymphoid tissues and blood and secreted high levels of IL‐21 to induce B‐cell antibody responses compared to CD4^+^CXCR5^−^ T cells.^16^, ^17^ Based on this concept, CD4^+^CXCR5^+^ helper T cells have become a target for elucidating the abnormal mechanisms of B‐cell responses in many autoimmune diseases. The effective roles of Tfh cells on the B‐cell responses were associated with the expression of ICOS or PD‐1 as well. ICOS signalling is known to be crucial for the generation of Tfh cells, germinal center formation and the production of high‐affinity antibodies.^11^, ^12^ Further studies have also reported that CD4^+^CXCR5^+^PD‐1^+^ T cells trigger high‐affinity antibody production and plasmablast differentiation following interactions with CD19^+^ B cells.^13^, ^18^ In many autoimmune diseases, CD4^+^CXCR5^+^PD‐1^+^ or CD4^+^CXCR5^+^ICOS^+^ cells have been found to be elevated, and these cells showed a positive correlation with disease severity and autoantibody titers.^18^, ^19^, ^20^, ^21^, ^22^ However, in recent years, PD‐1^+^ Tph cells lacking CXCR5 expression have also been reported to play a role in autoimmune B‐cell responses. These cells are suggested to express the CCR2 chemokine receptor, rather than CXCR5 expression, and to promote antibody production in these areas.^10^ These findings suggest that PD‐1 or ICOS molecules, rather than CXCR5, may play a more decisive role in regulating B‐cell responses and promoting autoantibody production. In the present study, we first sought to address this question by analysing the separate and combinational effects of these molecules on B‐cell responses. The results revealed that PD‐1 and also ICOS on T cells have an impact on B cells, independent of CXCR5 expression. Moreover, CXCR5^+^ helper T cells revealed a weak effect on B cells when they lacked PD‐1 or ICOS expression. However, the response of B cells to PD‐1 or ICOS expressing helper T cells was stronger in CXCR5 positive cells than in CXCR5 negative ones. Thus, the functional effectiveness of CXCR5^+^ cells in the Tfh phenotype on B‐cell responses was linked mainly to PD‐1 or also ICOS expression. While ICOS or PD‐1 expressing cells in the ‘Tph’ phenotype also induced B‐cell responses, they were less effective than Tfh cells. Although the effects of T‐cell subsets expressing PD‐1 or ICOS on B‐cell responses were demonstrated in this study, the lack of analysis of PD‐L1, PD‐L2 and ICOSL expression on B cells limits a more comprehensive and mechanistic interpretation of the observed functional differences. Evaluation of this axis in future studies may contribute to a better functional understanding of T–B‐cell interactions.

When the efficacy of Tfh (CXCR5^+^)‐ and Tph (CXCR5^−^)‐associated cell populations in AChR‐MG patients were compared with HC in co‐cultures containing both PD‐1^+^ Tfh and Tph cells, IgG, IL‐4 and IL‐17 productions were all higher. In assessing anti‐AChR‐IgG production, a prominent role of CXCR5^+^PD‐1^+^ cells was observed. However, CXCR5^−^PD‐1^+^ Tph cells also induced higher AChR‐IgG production, albeit weaker than in Tfh cells, indicating that CXCR5^+^PD‐1^+^ Tfh cells are the most potent population in autoantibody production, followed by PD‐1 expressing Tph cells in this process.

In MG, CD4^+^ T cells expressing PD‐1 are associated with enhanced B‐cell differentiation and antibody production rather than exhibiting a suppressive function. Consistent with the present results, a recent study reported the critical role of PD‐1 in regulating B‐cell memory and antibody production, and a reduction in memory B cells and impaired antibody responses in PD‐1 deficiency or inhibition.^23^ On the contrary, while PD‐1‐targeted immunotherapies in cancer are effective in enhancing immune responses, they can paradoxically lead to the emergence of autoimmune diseases such as MG in some patients.^24^, ^25^ This underscores the dual role of PD‐1 in both immune suppression and activation, highlighting the importance of understanding how this molecule functions in different settings. The immunosuppressive function of PD‐1 is generally mediated through its effects on Treg cells^26^ and is essential for maintaining immune tolerance.^27^ Anti‐PD‐1 therapy enhances the tumor immune response by reducing the suppressive effects of Treg cells in the tumor microenvironment.^28^ Blocking PD‐1 may disrupt Treg function, leading to excessive immune activation,^29^ which could potentially trigger the development of autoimmune diseases like MG during cancer treatments. However, the opposing effects observed in PD‐1 signalling across different T‐cell subsets emphasise the need for a better understanding of how PD‐1 modulates immune responses. Elucidating these mechanisms is a critical step toward optimising therapeutic strategies and understanding the pathogenesis of autoimmune diseases.

One limitation of the present study is the low frequency of CXCR5^+^ICOS^+^ cells, which prevented a comprehensive evaluation of the specific effects of ICOS expression in MG patients. The need for larger blood volumes for analysis restricted the ability to assess the distinct impact of ICOS expression separately from PD‐1.

Another limitation of this study is the lack of consideration for the expression kinetics of the cell subtypes. The dynamic changes in the expression of molecules such as PD‐1 and ICOS, as well as activation markers like CD40L and CD69, on Tfh and Tph cells during the 8‐day co‐culture with B cells were not assessed. This kinetics could provide more detailed insights into the temporal changes of Tfh and Tph cell activities influencing B‐cell responses.

Another significant limitation of this study was the measurement of IL‐21 as the more typical cytokine of both Tfh and Tph cells, which was constrained by the insufficient sensitivity of the assay method and the limited volume of culture supernatants.

Additionally, a limitation of the study is the age difference between the HC and MG groups, which may represent a potential confounding factor because of age‐related changes in immune function. This imbalance was mainly driven by practical constraints in recruiting healthy donors, as the isolation of rare T‐cell subsets (particularly CXCR5^+^PD‐1^+^ and CXCR5^+^ICOS^+^ populations) required substantially larger blood volumes from healthy individuals compared with MG patients.

In conclusion, PD‐1 expressing CD4^+^ helper T cells are associated with increased B‐cell helper activity and enhanced antibody responses in AChR‐MG. This association is more pronounced in CXCR5^+^ Tfh‐like cells, while CXCR5^−^ Tph cells also contribute to B‐cell responses. Overall, these findings suggest that PD‐1 expressing CD4^+^ T‐cell subsets contribute to dysregulated humoral immunity in MG.

## Methods

### Study group

The study included HC [*n* = 10, five men and five women, median age (range): 33 (25–35)] and AChR antibody‐positive, generalised and non‐thymoma AChR‐MG patients [*n* = 6, four men and two women, median age (range): 48 (18–81)]. Half of the HC and 34% of the patients were women. None of the AChR‐MG patients were receiving immunosuppressive therapy and had thymectomy at the time of blood sampling. According to the Myasthenia Gravis Foundation of America (MGFA) Clinical Classification, there were two patients with a score of 1, two with 2a and two with 3. The study was approved by the Istanbul Faculty of Medicine Ethics Committee. Written informed consent was obtained from all donors. The study was conducted in accordance with relevant ethical regulations, and all participant data were anonymised.

### Isolation of T and B cells from the peripheral blood

Peripheral blood mononuclear cells (PBMCs) were obtained using a Ficoll density gradient centrifugation method from donor bloods (40–60 mL). The cell pellet was resuspended in 1 mL medium containing 10% fetal bovine serum (FBS; PAN‐BioTech), 1% L‐glutamine (PAN‐Biotech) and 1% Penicillin–Streptomycin (PAN‐Biotech) in RPMI 1640 (Gibco). Live cell count was determined using trypan blue staining (BI; Biological Industries). Within the PBMCs, T cells were labelled with CXCR5 (PE, Clone: 51505; R&D), ICOS (Pe‐Cy7, Clone: ISA3; Invitrogen), PD‐1 (Clone: APC, Clone: EH12.2H7; Sony) and CD4 (FITC, Clone: RPAT4; Sony) antibodies and sorted into CXCR5^+^PD‐1^+^ICOS^−^, CXCR5^+^ICOS^+^PD‐1^−^, CXCR5^+^ICOS^−^PD‐1^−^, CXCR5^−^PD‐1^+^ICOS^−^, CXCR5^−^ICOS^+^PD‐1^−^ and CXCR5^−^ICOS^−^PD‐1^−^ helper T‐cell subsets using a cell sorter (BD FACSAria™ II) (Supplementary figure 1). From the same donors, B cells were labelled with CD19 (APC, Clone: HIB19; Sony) antibody, and CD19^+^ B cells were sorted (BD FACSAria II) as well the next day. The purity of the sorted cells was higher than 95%.

### T‐ and B‐cell co‐culture

Flat‐bottom 96‐well culture plates were coated with anti‐CD3 and anti‐CD28 antibodies (10 and 5 μg mL^−1^, respectively) overnight. Sorted helper T‐cell subgroups according to CXCR5, ICOS and PD‐1 expression (50 000 cells per well) were added to these wells and incubated at 37°C with 5% CO_2_ for 1 day. As a control, only medium was added to a single well, where CD19^+^ B cells will be added later.

After 24 h, CD19^+^ B cells of the same donors were isolated from PBMCs using cell sorter (the FACSAria II) and these cells were also added to each well at a concentration of 100 000 cells per well. After 8 days of the co‐culture period, the supernatants were collected and stored at −80°C for subsequent measurements.

### Phenotypic analyses of plasmablast cells

After the co‐culture period, cells were collected and centrifuged at 440 rpm for 10 min at 4°C. The pelleted cells were then stained with antibodies against CD4 (PE‐Cy7, Clone: RPAT4; Sony), CD19 (APC, Clone: HIB19; Sony), CD27 (PE, Clone: O323; BioLegend) and CD38 (PerCP‐Cy5.5, Clone: HB‐7; BioLegend) at room temperature for 30 min and analysed using a flow cytometer. Within the CD19^+^ B‐cell population, the CD27^+^CD38^+^ PB were quantified (Supplementary figure 2). Flow cytometry data were analysed using the FlowJo software (FlowJo LLC, USA).

### Measurement of total IgG and anti‐AChR‐IgG concentrations

In co‐culture supernatants, IgG levels were measured using a total IgG ELISA Kit (Invitrogen). Specific anti‐AChR antibody concentrations were determined by an AChR‐IgG ELISA kit (EUROIMMUN), according to the manufacturer's instructions. The detection limit for the total IgG and for the AChR‐IgG was 0.24 ng mL^−1^ and 0.11 nmol L^−1^.

### Measurement of cytokine concentrations

IL‐21, IL‐4 and IL‐17 concentrations in co‐culture supernatants at Day 8 were measured using a cytometric bead array kit (BD Biosciences) according to the manufacturer's instructions. The sensitivities of the tests were 34.3 pg mL^−1^ for IL‐21, 26.1 fg mL^−1^ for IL‐17 and 144.4 fg mL^−1^ for IL‐4.

### Statistical analysis

To assess the differences between co‐cultures as well as patient and control groups, non‐parametric statistical tests (pairwise comparisons of related samples and Mann–Whitney U‐tests) were used. Statistical significance was defined as *P* < 0.05. Figures were generated using GraphPad Prism 10, and the results are presented as medians with interquartile ranges.

## Author contributions

**Hacer Durmuş:** Conceptualization; formal analysis; data curation. **Merve Çebi:** Conceptualization; data curation; writing – original draft; visualization; methodology. **Yeşim Parman:** Writing – review and editing; supervision. **Güher Saruhan‐Direskeneli:** Conceptualization; methodology; writing – review and editing; project administration; supervision; data curation; investigation; visualization. **Arman Çakar:** Conceptualization; data curation.

## Conflict of interest

The authors declare no conflict of interest.

## Supporting information


Supplementary figure 1‐2

## Data Availability

The data that support the findings of this study are available from the corresponding author upon reasonable request.
