# Supplementary material for: Effect of CXCR5, PD‐1 and ICOS on B‐cell responses and relevance to myasthenia gravis
Source: Clin Transl Immunology. 2026 Jun 21;15(6):e70112. doi: 10.1002/cti2.70112 (PMC13283754; doi:10.1002/cti2.70112)
Supplement: Supplementary file 1 — Supplementary figure 1‐2 [file CTI2-15-e70112-s001.docx]

**Supplementary data:**

**Sorting of helper T cells based on CXCR5, PD-1, and ICOS expression**

Helper T cells within PBMCs were labeled with CXCR5 (PE, R&D), ICOS (Pe-Cy7, Invitrogen), PD-1 (APC, Sony), and CD4 (FITC, Sony) antibodies, and were sorted into CXCR5**^+^**PD-1**^+^**ICOS**^-^**, CXCR5**^+^**ICOS**^+^**PD-1**^-^**, CXCR5**^+^**ICOS**^-^**PD-1**^-^**, CXCR5**^-^**PD-1**^+^**ICOS**^-^**, CXCR5**^-^**ICOS**^+^**PD-1**^-^**, and CXCR5**^-^**ICOS**^-^**PD-1**^-^** helper T cell subsets using a BD FACSAria™ II cell sorter. (Supplementary Fig. 1).


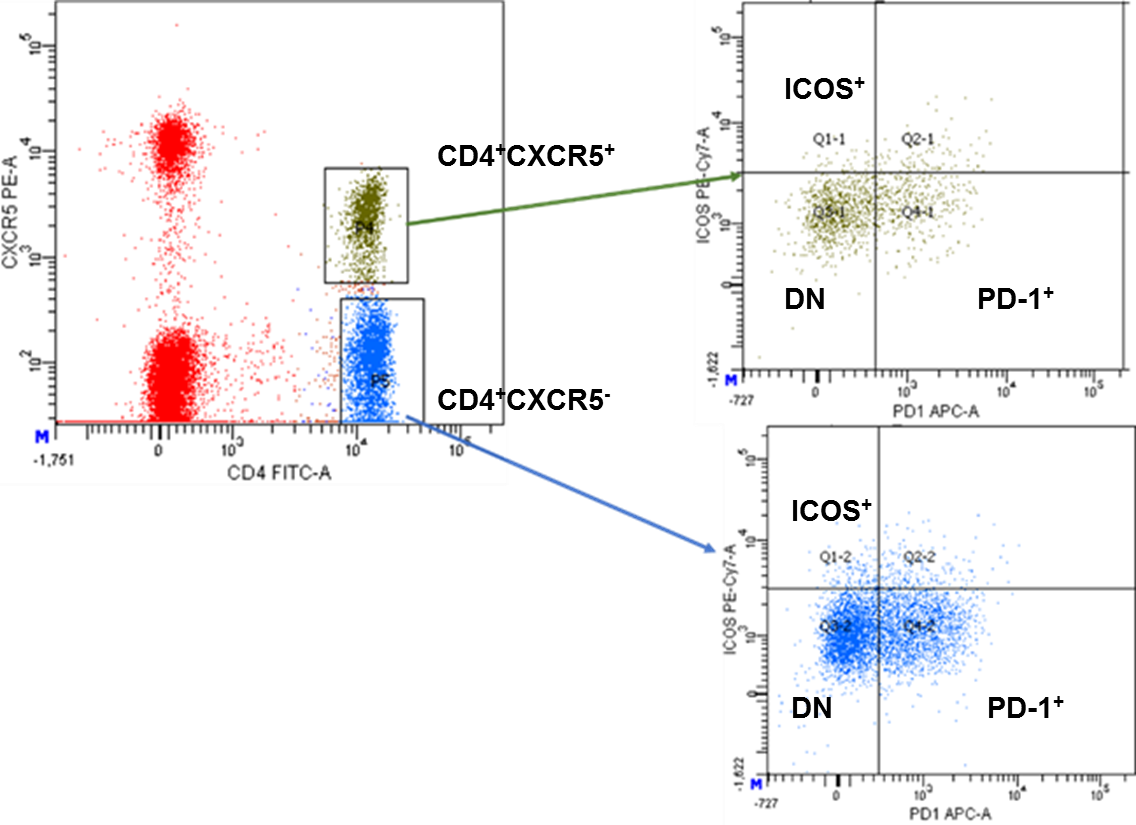


**Supplementary Fig. 1:** PBMCs were labeled with CD4, CXCR5, ICOS, and PD-1 antibodies. CD4**^+^**CXCR5**^+^** or CD4**^+^**CXCR5**^-^** cells within the lymphocyte gate were identified, and within these populations, ICOS**^+^**PD-1**^-^**, ICOS**^-^**PD-1**^+^**, and ICOS**^-^**PD-1**^-^** (Double Negative, DN) cells were gated and sorted using a BD FACSAria™ II system.


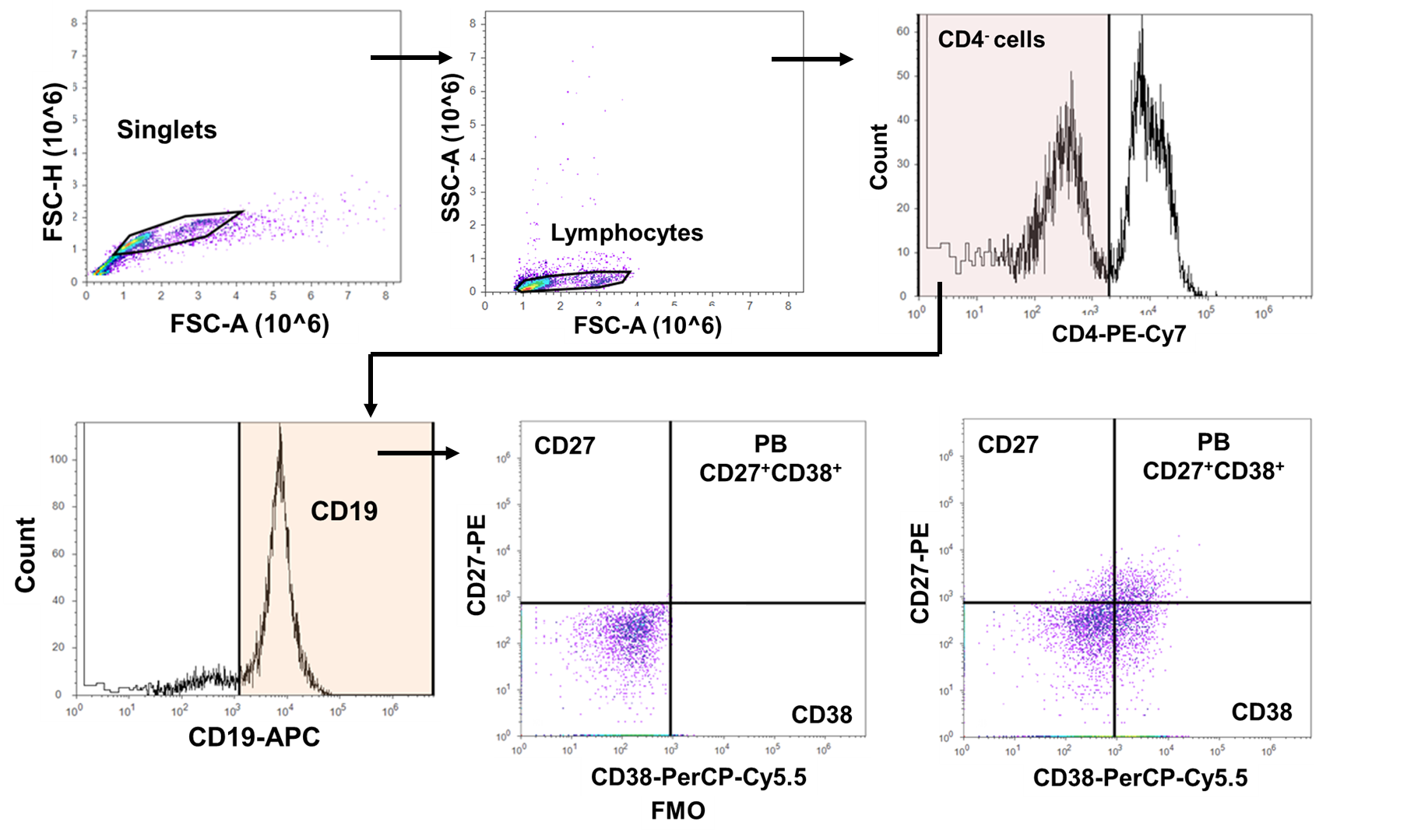


**Supplementary Fig.2: Gating strategy of plasmablast population**

CD27^+^CD38^+^ cells within CD19^+^ B cells have been defined as plasmablast cells.
